# Unravelling the distinct effects of VHL mutations and chromosome 3p loss in clear cell renal cell carcinoma: Implications for prognosis and treatment

**DOI:** 10.1002/ctm2.70465

**Published:** 2025-09-25

**Authors:** Xiang Wang, Jian‐Rong Li, Naail Raed Chowdhury, Lang Wu, Cheng Chao

**Affiliations:** ^1^ Institute for Clinical and Translational Research Baylor College of Medicine Houston Texas USA; ^2^ Graduate Program in Quantitative and Computational Biosciences Baylor College of Medicine Houston Texas USA; ^3^ Section of Epidemiology and Population Sciences Department of Medicine Baylor College of Medicine Houston Texas USA; ^4^ Population Sciences in the Pacific Program (Cancer Epidemiology) University of Hawaii Cancer Centre Honolulu Hawaii USA; ^5^ Dan L. Duncan Comprehensive Cancer Centre Baylor College of Medicine Houston Texas USA

1

Dear Editor,

In this study, we delineated the distinct transcriptomic effects of VHL mutation and chromosome 3p (chr3p) loss, revealing that chr3p loss is specifically associated with immune suppression in clear cell renal cell carcinoma (ccRCC). Furthermore, we developed driver genomic aberration (DGA) gene signatures that demonstrate superior performance in predicting both patient prognosis and treatment response compared to traditional mutation‐based approaches.

Renal cell carcinoma (RCC) accounts for 80%–85% of all primary kidney cancers, with ccRCC being the most common subtype (∼75%).^1^ In 2023, ∼82 000 new RCC cases and ∼15 000 deaths were reported in the U.S. Despite surgery being curative for localised disease, ∼33% of patients relapse, and those with metastatic disease (∼15%) have a poor prognosis.^1^ Despite treatment advances, significant variability in outcomes highlights the need for reliable molecular biomarkers to guide the treatment. Large‐scale genomic studies such as the Cancer Genome Atlas (TCGA) have shown that the VHL gene is frequently inactivated in ccRCC through mutation or chr3p deletion.^2^ However, the prognostic and therapeutic relevance of VHL mutations and chr3p loss remains controversial.

We examined the most frequently mutated genes in the TCGA KIRC dataset.^2^ The *VHL* gene exhibited the highest mutation rate (52%), followed by *PBRM1* (31%), *SETD2* (11%) and *BAP1* (5%) (Figure 1A). Copy number analysis revealed that chr3p loss occurred in 27% of patients, and most loss events, interestingly, encompassed these four genes. (Figure 1B). This genomic configuration is largely unique to ccRCC among TCGA cancer types (Figure S1A).

**FIGURE 1:**
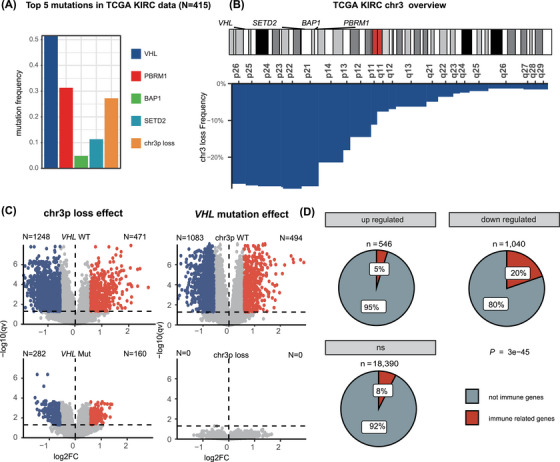
Clear cell renal cell carcinoma (ccRCC) driver mutations and their effects on the transcriptomic profile. (A) The top five most frequent genomic aberrations in ccRCC patients from the Cancer Genome Atlas (TCGA) dataset. (B) Landscape of chr3p loss in ccRCC patients in the TCGA dataset. (C) Volcano plots illustrating the transcriptomic effects of chr3p loss in *VHL* wild‐type and *VHL*‐mutated ccRCC patients (left) and transcriptomic effects of VHL mutation in chr3p wild‐type (WT) and chr3p loss ccRCC patients (right). (D) Pie chart showing the proportion of immune‐related genes among upregulated, downregulated, and non‐regulated genes associated with chr3p loss.

Mutation rates of these genes were consistent across tumour stages, indicating early tumourigenic roles (Figure S1B). To investigate the transcriptomic effects of *VHL* mutation and chr3p loss, we stratified TCGA‐KIRC data by these aberrations and identified differentially expressed genes. In *VHL*‐WT tumours, chr3p loss led to 1719 differentially expressed genes (DEGs; FDR < .05, |log_2_FC| > 1.5), while in tumours with intact chr3p, *VHL* mutation resulted in 1577 DEGs. However, in the presence of *VHL* mutation, chr3p loss still induced 442 DEGs, whereas *VHL* mutation had no significant transcriptomic impact in chr3p‐loss tumours, indicating that chr3p loss exerts a dominant regulatory effect (Figure 1C). Notably, immune‐related genes were significantly enriched in genes that were downregulated in chr3p‐loss tumours (Figure 1D), and GSEA analysis confirmed the suppression of immune pathways (Figure S2).

Given that immune gene suppression associated with chr3p loss, we explored its impact on the tumour immune microenvironment (TIME). Using previously reported data,^3^ we discovered that leukocyte and lymphocyte infiltration levels in TCGA‐KIRC were significantly lower in chr3p‐loss versus chr3p‐WT tumours (*p* < .01), whereas *VHL* mutation had no significant impact on immune infiltration metrics (Figures 2A,B).

**FIGURE 2:**
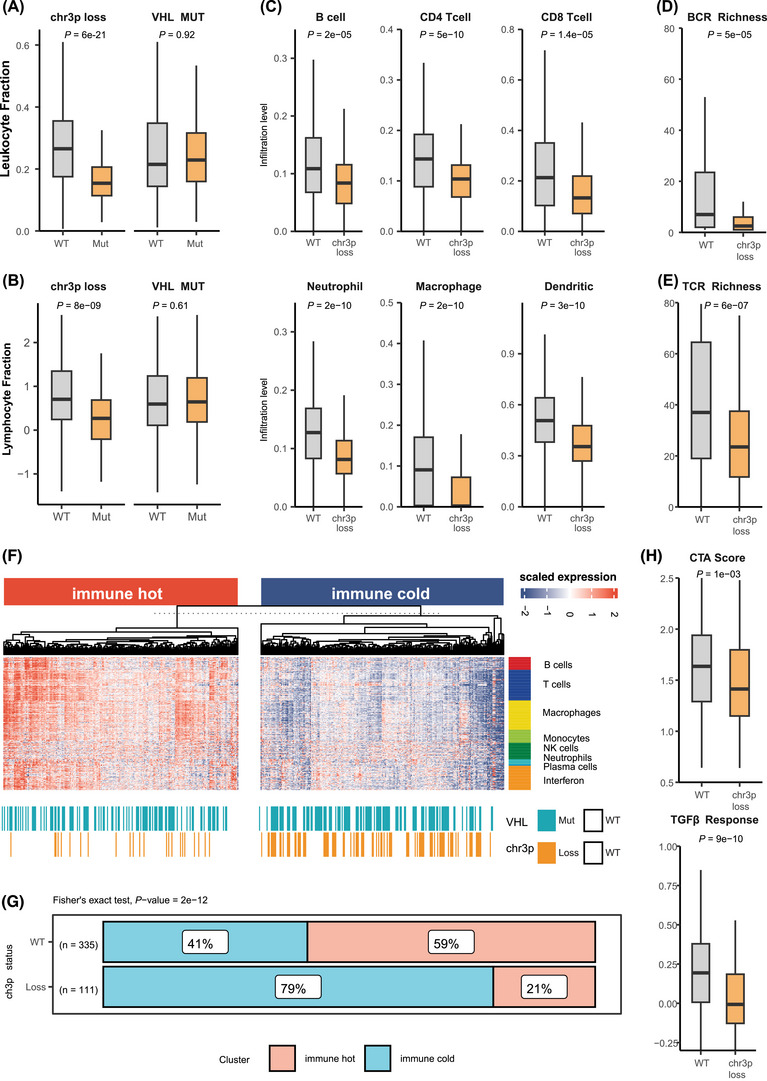
Immune landscape of *VHL* mutation and chromosome 3p (chr3p) loss in clear cell renal cell carcinoma (ccRCC) tumours. (A‐B) Boxplots comparing (A) leukocyte fractions and (B) lymphocyte fractions between chr3p‐loss and VHL‐mutated patients versus their wild‐type counterparts. (C) Boxplot showing the comparison of TIMER‐estimated immune cell fractions between chr3p‐deleted patients and wild‐type patients. (D, E) Boxplot comparing (D) B cell and (E) T cell receptor expression between chr3p‐deleted and wild‐type patients. (F) Heatmap illustrates the expression of immune‐related genes in ccRCC patients, categorised into immune “hot” and immune “cold” phenotypes. (G) Barplot depicting the proportion of immune “hot” and immune “cold” patients within the chr3p wild‐type and chr3p‐loss groups. (H) Boxplot comparing chr3p‐associated immune functions, including cancer‐testis antigen (CTA) scores and TGF‐beta response, between wild‐type and chr3p‐deleted groups.

Using the TIMER algorithm,^4^ we estimated immune cell infiltration levels and observed that chr3p‐loss tumours had significantly reduced infiltration of B cells, CD8+ and CD4+ T cells, macrophages, dendritic cells and neutrophils (Figure 2C). We also quantified BCR and TCR richness from RNA‐seq reads and found both significantly diminished in chr3p‐loss tumours, consistent with the immune suppression phenotype (Figures 2D,E). We next performed unsupervised clustering using expression of immune cell marker genes (Table S1) to classify tumours into immune “hot” and “cold” clusters. Chr3p‐loss tumours were significantly enriched in the immune‐cold cluster (79% vs. 41% in chr3p‐WT; *p* = 9e‐12, Fisher's exact test), while *VHL*‐mutant tumours were evenly distributed, indicating that chr3p loss, not *VHL*, drives immune suppression (Figures 2F,G). To better understand this, we did stratified analysis, and chr3p loss alone showed reduced immune infiltration, which is not observed in *VHL* mutation only samples, suggesting a possible contribution from other genes from the region, such as *BAP1* (Figure S3). Further supporting this, chr3p‐loss samples exhibited significantly lower cancer‐testis antigen (CTA) scores and reduced TGF‐β pathway activity (Figure 2H). Both measures reflect diminished immune activity and T‐cell function.

Despite the suppressed TIME caused by chr3p loss and the drastic transcriptomic change caused by the mutation, the prognostic significance and ability of the mutation status of chr3p loss and *VHL* remain contentious.^5^, ^6^ We therefore applied a transcriptomic signature‐based approach by developing DGA gene signatures using TCGA mutation and copy number alteration (CNA) data to quantify the downstream effects of these alterations (Table S2). The *VHL* mutation signature achieved an AUC of  0.79 in classifying *VHL* mutation status and was validated in external datasets, including the Gordan cohort^7^ (Figure 3A) and CCLE data (Figure 3B), showing that the DGA signatures can capture genomic aberration dysregulated transcriptional activity.

**FIGURE 3:**
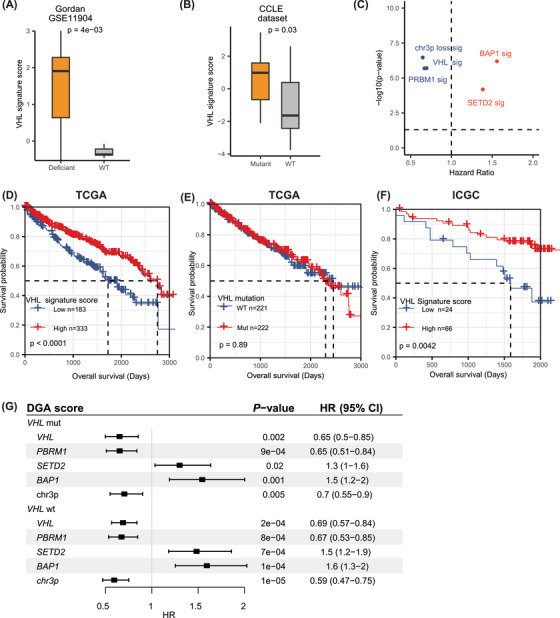
Driver genomic aberration (DGA)‐associated signatures predict clear cell renal cell carcinoma (ccRCC) patient prognosis. (A) The VHL signature predicts patient genotypes in the Gordan dataset. (B) Boxplot showing significant differences in VHL signature expression between wild‐type and mutant cell line data from the Cancer Cell Line Encyclopedia (CCLE). (C) Volcano plot demonstrating that all five DGA‐associated gene signatures are significantly associated with prognosis in the Cancer Genome Atlas (TCGA) ccRCC patients. (D, E) Kaplan‐Meier plots illustrating stratified analyses of (D) mutation signature scores and (E) VHL mutation status in relation to overall survival in TCGA ccRCC patients. (F) Kaplan‐Meier plot showing the relationship between VHL signature scores and overall survival in ccRCC patients from the ICGC EU RCC cohort. (G) Forest plot illustrating the correlation between DGA‐associated signatures and overall survival, analysed using Cox regression in both VHL wild‐type and mutated patient groups.

We next evaluated the prognostic value of the DGA signatures. chr3p loss, *VHL*, *PBRM1*, *SETD2*, and *BAP1* mutation derived signature is significantly associated with patient survival. Specifically, higher scores for *PBRM1* and chr3p loss were linked to better prognosis, whereas elevated *SETD2* and *BAP1* signature scores correlated with poorer survival (Figure 3C). More specifically, while *VHL* mutation status alone did not show significant prognostic value (Figure 3D), the VHL signature score was strongly associated with improved overall survival in the TCGA cohort (Figure 3E), underscoring the utility of transcriptomic signatures in capturing functional pathway disruption beyond mutational status. This was replicated in the ICGC EU RCC cohort,^8^ where patients with high VHL scores had significantly longer survival (Figure 3F). As our VHL signature score reflects mutation‐regulated transcriptomic dysregulation, it provides a continuous measure of such dysregulation. To further validate its prognostic utility, we performed a multivariate Cox regression survival analysis for both VHL wild‐type and mutated patients (Figure 3G). The results demonstrate that the DGA signature scores remain significant predictors of patient outcomes, highlighting oncogenic pathway activities as main contributing factors to prognosis in ccRCC.

Given that sunitinib targets VEGFR‐mediated angiogenesis, and *VHL* regulates hypoxia and angiogenesis, we investigated the correlation between VHL signature scores and tumour angiogenesis activity. Using an angiogenesis gene set, we found that angiogenic activity is significantly higher in VHL signature high samples (Figure 4A).

**FIGURE 4:**
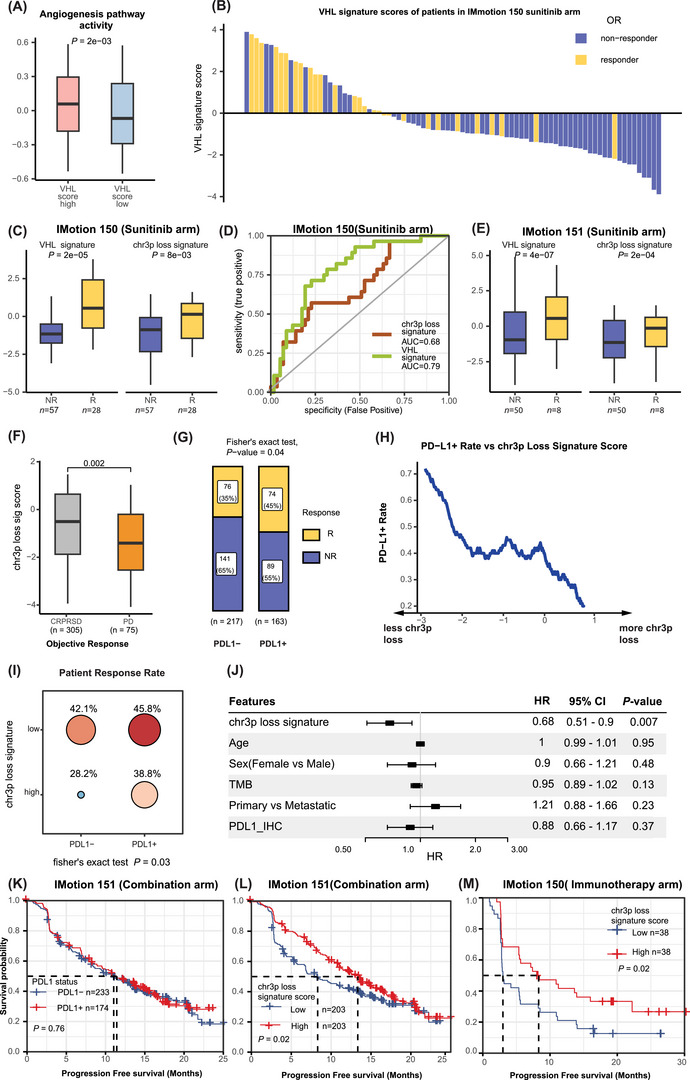
Driver genomic aberration (DGA) signature scores predict therapy response. (A) Boxplot showing that patients with high VHL signature scores exhibit higher angiogenesis pathway activity compared to those with low scores. (B) Waterfall plot illustrating the relationship between VHL signature scores and treatment response. (C) Boxplot comparing VHL signature and chromosome 3p (chr3p) loss signature scores between responders and non‐responders in the IMmotion150 trial. (D) Receiver operating characteristic (ROC) curve evaluating the predictive performance of VHL signature and chr3p loss signature scores for treatment response. (E) Boxplot comparing VHL signature and chr3p loss signature scores between responders and non‐responders in the IMmotion151 trial. R: Responder; NR: Non‐responders (F) Boxplot comparing chr3p loss signature scores among patient groups with complete response (CR), partial response (PR), stable disease (SD), to progressive disease (PD). (G) Barplot illustrating the percentage of responders and non‐responders in PD‐L1‐negative (PD‐L1‐) and PD‐L1‐positive (PD‐L1+) ccRCC patient groups. (H) Line plot showing the relationship between PD‐L1 status and chr3p loss signature scores. (I) Dot plot categorising patient response rates based on PD‐L1 status and chr3p loss signature scores. (J) Forest plot presenting the relationship between chr3p loss signature scores and progression‐free survival (PFS) in ccRCC patients, along with clinical variables like age, sex, TMB status, metastatic status and PDL1 status. (K‐L) Kaplan‐Meier (KM) plots showing the relationship of (K) PD‐L1 status and (L) chr3p loss signature scores with PFS in the IMmotion151 dataset. (M) Kaplan‐Meier plot depicting the relationship between chr3p loss signature scores and PFS in the IMmotion150 dataset.

In the IMmotion150 trial,^9^ sunitinib responders had higher *VHL* and chr3p signature scores than non‐responders (Figure 4B,C). Receiver operating characteristic analysis showed that the VHL signature predicted response with an AUC of  0.79, and the chr3p loss signature with an AUC of  0.68 (Figure 4D). These findings were validated in IMmotion151, further demonstrating the predictive capacity of the VHL signature for VEGF‐targeted therapy response (Figure 4E).

To evaluate the utility of the chr3p loss signature in immunotherapy, we analysed patient outcomes in the IMmotion151 (combination therapy) and IMmotion150 (atezolizumab monotherapy) trials.^9^ Patients with higher chr3p scores were more likely to experience CR, PR, or SD, while low scores were associated with PD (Figure 4F). PD‐L1 expression (Figure 4G) and tumour mutational burden (TMB, Figure S4) showed only weak associations with response. Interestingly, PD‐L1 positivity inversely correlated with chr3p score (*r* = –0.31), suggesting that the chr3p signature scores provide independent insights (Figure 4H). Combining PD‐L1 and chr3p signature score (top25% vs rest) improved response stratification: PD‐L1‐, chr3p‐high patients showed lower response rates than all other groups (*p* = .03, Figure 4I). Multivariable Cox regression for progression‐free survival (PFS) confirmed that the chr3p signature was the only significant predictor (HR = .85, *p* = .001), while PD‐L1 was not (Figure 4J). High chr3p scores were associated with longer PFS in both IMmotion151 and IMmotion150 datasets (Figure 4K,L). In the latter, despite a smaller sample size, chr3p remained predictive (HR = .74, *p* = .02, Figure 4M). To further validate the result, we have applied it to CheckMate data and observed chr3p signature is protective regarding patient overall survival ^10^(Figure S5).

In summary, we demonstrate that chr3p loss, rather than *VHL* mutation, drives immune suppression in ccRCC. Although we have not experimentally validated the mechanism, the overall low genome instability of ccRCC and a lack of association between genome instability and immune response point to chr3p loss being the cause of such immune suppression. Using transcriptomic‐based DGA signatures, we identify distinct impacts of these alterations on prognosis and treatment response. The *VHL* and chr3p signatures outperform mutation status in predicting patient survival and guiding target and immunotherapy. Although our results are validated across independent clinical datasets, we acknowledge the absence of experimental validation and prospective trials as a limitation due to the scope of this paper. The scores can be applied to the clinical setting with a threshold simply using zero or better stratification based on the patient population in clinical testing. Our findings provide a clinically relevant framework for stratifying ccRCC patients and advancing precision oncology and guiding clinical trial design by paring immune checkpoint therapy with VEGFR inhibitors.

## AUTHOR CONTRIBUTIONS

Cheng Chao conceived the project. Cheng Chao and Xiang Wang obtained the data. Xiang Wang and Cheng Chao developed the methods. Xiang Wang and Cheng Chao performed computational analyses. Xiang Wang and Cheng Chao wrote the manuscript. Xiang Wang and Cheng Chao interpreted the results. Xiang Wang and Jian‐Rong Li made figures. Cheng Chao supervised the project. All authors critically reviewed the content. All authors read and approved the final manuscript.

## CONFLICT OF INTEREST STATEMENT

The authors declare no conflict of interest.

## FUNDING INFORMATION

This work is supported by the Cancer Prevention Research Institute of Texas (CPRIT) (RR180061) and the National Cancer Institute of the National Institutes of Health (1R01CA269764).

## ETHICS STATEMENT

Not applicable.

## Supporting information



Supporting Information

Supporting Information

Supporting Information

## Data Availability

The data that support the findings of this study are openly available through the Gene Expression Omnibus (GEO) database, TCGA‐KIRC (http://firebrowse.org), CCLE database (https://depmap.org/portal/download/all/). ICGC and Clinical trial data from EGAD00001004183 and EGAS0000100435 are available on request.^9^ Checkmate data can be accessed from Braun et al.^10^
