# Supplementary material for: Unravelling the distinct effects of VHL mutations and chromosome 3p loss in clear cell renal cell carcinoma: Implications for prognosis and treatment
Source: Clin Transl Med. 2025 Sep 25;15(10):e70465. doi: 10.1002/ctm2.70465 (PMC12463683; doi:10.1002/ctm2.70465)
Supplement: Supplementary file 2 — Supporting Information [file CTM2-15-e70465-s002.docx]

**Supplementary Figures:**

**Supplementary Figure 1.** **Genomic characteristic of ccRCC**

**A.** Frequency of VHL mutations (left) and chr3p loss (right) across different cancer types in TCGA cohorts with over 100 patients per cancer type. **B.** Mutation rates of the top five driver genomic aberrations at different stages of ccRCC.

**
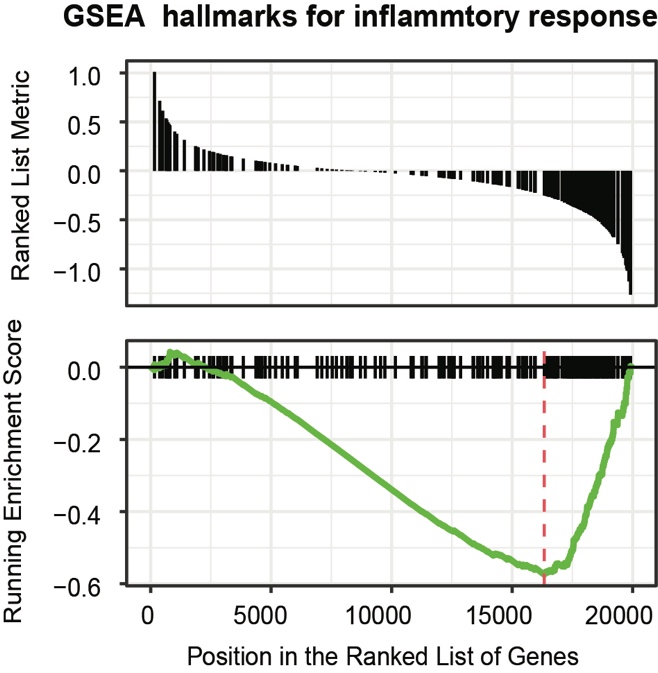
**

**Supplementary Figure 2.** **Transcriptomic Hallmark Analysis of Chr3p Loss in ccRCC**
Gene Set Enrichment Analysis (GSEA) plot illustrating the ranked list metric and running enrichment score for inflammatory response-related genes in chr3p-loss samples

**
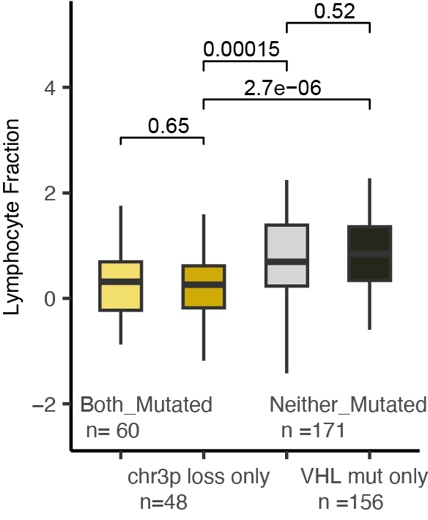
**

**Supplementary Figure 3. Stratified analysis of chr3p loss and VHL mutation effect on TIME**

Boxplots comparing lymphocyte fractions between chr3p-loss and VHL-mutated patients versus sample with both mutation and neither mutation.

**Supplementary Figure 4.** **Association Between Tumor Mutational Burden (TMB) and Patient Response in ccRCC**
A bar plot illustrating the distribution of patients with **high** and **low tumor mutational burden (TMB) status,** along with the proportion of responders and non-responders to therapy.

**Supplementary Figure 5. Chr3p loss signature is protective toward PD-1 checkpoint therapy in ccRCC patients.**

Forest plot presenting the relationship between chr3p loss signature scores and overall survival (OS) in ccRCC patients, along with clinical variables like age, sex, and metastatic status.
