# Supplementary material for: Unravelling the distinct effects of VHL mutations and chromosome 3p loss in clear cell renal cell carcinoma: Implications for prognosis and treatment
Source: Clin Transl Med. 2025 Sep 25;15(10):e70465. doi: 10.1002/ctm2.70465 (PMC12463683; doi:10.1002/ctm2.70465)
Supplement: Supplementary file 3 — Supporting Information [file CTM2-15-e70465-s001.docx]

**Supplementary Methods:**

**Data Collection and Processing**

For our study, we used Level 3 processed data from　TCGA　focusing on Kidney Renal Clear Cell Carcinoma (KIRC)[1]. This comprehensive dataset was obtained from FireBrowse (http://firebrowse.org). It encompasses various sequencing datasets from a total of 513 patients, including RNA-sequencing data for all patients, somatic mutation data for 448 patients, and copy number variation data. Additionally, proteomics data paired with extensive clinical information—such as age, sex, overall survival, and treatment details—were also included. This dataset enabled an in-depth examination of the genomic and transcriptomic influence of *VHL* gene in the context of ccRCC. Furthermore, we used this data to construct *VHL*-specific and chr3p genomic signatures, which were essential for subsequent analyses.

To validate our findings, we sourced additional transcriptomic and clinical data for kidney cancer from two major platforms:

GEO Dataset: GSE11904 – Accessed through the Gene Expression Omnibus (GEO), this dataset contains microarray expression data from 57 renal cell carcinoma (RCC) samples.[2] Each sample is accompanied by clinical data and *VHL* genotype information, providing a robust foundation for comparative analyses. To preprocess the raw microarray data, we applied the Robust Microarray Analysis (RMA) method for background correction, followed by quantile normalization to minimize inter-sample variations. Finally, probe set readings were consolidated into gene-level expression values to enable comprehensive downstream bioinformatics evaluations.

**ICGC Data**: From the International Cancer Genome Consortium (ICGC)[3], we utilized the RECA-EU project dataset, which includes RNA-sequencing data and clinical details from 91 patients. This dataset was retrieved from the ICGC data portal, featuring vital information such as patient survival times. The RNA-seq data from this cohort provided a secondary layer of validation, reinforcing the robustness of our study’s conclusions.

**IMMotion data:**  IMmotion151 (NCT02420821) is a multicenter, open-label, Phase 3, randomized controlled trial of atezolizumab plus bevacizumab (n=454) vs. sunitinib (n=461) in patients with previously untreated advanced renal cell carcinoma. IMmotion150 (NCT01984242) is the Phase 2 clinical trial of study. Patient clinical data and gene expression data were requested and downloaded after request under the accession number of EGAD00001004183 and EGAS00001004353[4].

**Cell line expression mutation data:**

The Drug sensitivity dataset was downloaded from the Cancer Cell line Encyclopedia (CCLE) database (<https://depmap.org/portal/download/all/>). Here, a total of 31 Renal Cell Line data were downloaded with matching expression and mutation information.

**Definition of Driver Mutations and Patient Classification:**

For the purpose of identifying driver mutations within our study, we analyzed whole-exome sequencing (WES) data that was previously processed from TCGA. Specifically, we focused on non-silent mutations. To ensure the robustness of our analysis, we excluded genes that are exceptionally long, as their high mutation rates could skew the results.

We established a threshold for identifying driver mutations based on their frequency within the study population. Genes were classified as harboring driver mutations if non-silent mutations occurred in more than 10% of the patient samples analyzed. We then cross-referenced the identified mutations with known cancer mutations listed in the Catalogue of Somatic Mutations in Cancer (COSMIC) to ensure robustness [5].

**Chromosomal Band Analysis and CNA Determination:**

We utilized CNA segment files from 513 Kidney Renal Clear Cell Carcinoma (KIRC) samples, which were retrieved from FireBrowse ([http://firebrowse.org](http://firebrowse.org/)). These files enumerate genomic regions characterized by significant CNA. To quantitatively assess each CNA segment, we calculated the segment mean as log₂(copy number/2). In this scale, a value of 0 indicates a normal copy number, whereas positive and negative values indicate genomic amplification and deletion, respectively.

To identify clinically significant CNA regions, we selected segments where the absolute mean values exceeded 0.5. This threshold was chosen to ensure the reliability of the data by focusing on alterations that are more likely to influence cellular phenotypes.

Following the identification of significant CNA segments, we analyzed the copy number status of specific chromosomal bands. A chromosomal band was classified as gained or lost if it overlapped by more than 50% with any significant CNA segment. Notably, chromosomal bands on the Y chromosome were excluded from this analysis due to their unique biological implications and less frequent involvement in KIRC.

Additionally, we normalized deletion frequencies to account for the differential detection sensitivity between deletion and amplification events. This normalization involved multiplying the total number of detected amplification events by the ratio of deletion to amplification events, providing a more balanced view of the genomic landscape across the KIRC samples.

**Defining DGA signature**

DGA signatures were defined by comparing the differential expression of genes between mutant and wild-type samples while considering confounding variables using TCGA KIRC RNA-seq data. Each driver genomic aberration is built separately by defining these sample groups. Samples containing synonymous driver mutations were assigned to the wild-type group.

$Y=\alpha+\sum_{i=1}^{m} \beta_{i}X_{i}+\sum_{j=1}^{n} \gamma_{j}Z_{j}$

Y is the expression level of the gene; X_i_ is the indicator function for the genomic event i (X_i_=1 for samples with the event i, and 0 otherwise); Z_j_ is the clinical variable j to be adjusted; m and n are the number of genomic events and clinical variable to be considered in the model. By applying these models to the TCGA KIRC data, we estimated the coefficients (β-values) and their statistical significance (*P*-value) for all genes. Second, given (β, p) values for all genes, we defined the DGA gene signature using a pair of weight profiles, w+ and w−, that assigned all genes two values in the following way: For gene i, $W_{i}^{+}=-log\left( p_{i} \right)I\left( \beta_{i}>0 \right)$and $W_{i}^{-}=-log\left( p_{i} \right)I\left( \beta_{i}<0 \right)$. To avoid extreme values, the weights were trimmed at 10, and then transformed into a value within [0,1], by subtracting the minimum value and then dividing by the range, by subtracting the minimum value and then dividing by the range. If a gene i is more significantly up-regulated in mutant versus wild-type samples, it will be associated with a higher $W_{i}^{+}$and $W_{i}^{-}$of zero. Conversely, a more significantly down-regulated gene will be associated with a higher $W_{i}^{-}$and $W_{i}^{+}$of zero.

**Calculating sample specific DGA signature score**

Given the expression of a sample, sample-specific DGA signature score is calculated using BASE algorithm that has been described previously [6]. A bigger DGA signature score would represent an increase in the mutation-dysregulated oncogenic pathway activity, and conversely, a lower signature means that the samples' expression shows little impact from the oncogenic pathway activity dysregulated by the specific mutation.

**Immune Cell Infiltration Analysis in ccRCC Tumors**

Our study utilized immune cell infiltration data which was initially computed and compiled as described in the seminal work by Thorsson et al [7].To further investigate the Tumor Immune Microenvironment (TIME) of clear cell renal cell carcinoma (ccRCC) tumors, we applied Tumor IMmune Estimation Resource (TIMER) algorithm to the TCGA dataset [8]. The algorithm uses statistical methods to evaluate the cell populations within different types of cancer with clinical data

In this study, our focus was on quantifying the infiltration levels of various immune cells which are pivotal in tumor immunology. These cells include leucocytes, lymphocytes, B cells, CD4 cells, CD8 T cells, macrophages, and neutrophils.

**ccRCC tumor Microenvironment Classification**:

To explore the tumor immune microenvironment of ccRCC tumors, we utilized a set of immune cell marker genes previously validated in the scientific literature [9]. This set includes markers for B cells, macrophages, monocytes, neutrophils, plasma cells, and interferon-responsive cells (Supplementary Table 1). We extracted and normalized the expression data for these markers from the TCGA Kidney Renal Clear Cell Carcinoma (KIRC) expression dataset. We then used the “*ComplexHeatmap”* module in R to generate a heatmap for the resultant expression data [10].

To classify the tumor samples based on their immune profiles, we applied hierarchical clustering in the ComplexHeatmap module and divided the resulting tree into two. This clustering resulted in the partitioning of the samples into two distinct groups. Samples that showed high expression levels of the selected immune cell markers were classified as 'immune hot'. Conversely, samples with low expression of these markers were designated as 'immune cold', indicating a relative lack of immune cell presence.

**Differential Expression Analysis**

The Student's T-test was utilized to determine differentially expressed genes between two tumor sample groups (e.g., *VHL* mutated samples vs. *VHL* wild-type samples). The resulting *P*-values were adjusted for multiple comparisons using the False Discovery Rate (FDR) method, with a significance threshold set at FDR < 0.05. We then adopted a 1.5-fold change as the threshold to identify genes that are significantly up or down-regulated between the two groups.

**Gene pathway enrichment analysis (GSEA)**

GSEA analysis was performed on the differentially expressed genes across various patient populations stratified by mutation status or DGA signature scores [11]. The Hallmark gene sets were utilized to identify enriched gene pathways. Additionally, GSVA was applied to examine specific pathway activity differences between the two patient groups[12].

**Statistical Analysis**

We used univariate and multivariate Cox regression models to investigate the association between the DGA signature and patient prognosis. These models were adjusted for potential confounding variables, including age, sex, and tumor stage. The analysis was performed using the “*coxph”* function from the R *“survival”* package. Survival differences between patient groups were visualized with Kaplan–Meier (KM) plots, with p-values calculated using the log-rank test. The KM curves were created using the “*survfit”* function, and the survival plots were generated with “*ggsurvplot”*.

**AUC Score Calculation**

The Area Under the Curve (AUC) scores for each gene's prediction of immunotherapy response were calculated using gene expression versus drug response data, employing the “*ROCR”* package. Additionally, the AUC scores for signature scores in predicting targeted therapy response were determined by applying a cut-off to the signature scores for patients, with True Positive Rate (TPR) and False Positive Rate (FPR) computed from the selected cut-off. The AUC scores were then calculated using these TPR and FPR values.

1. Ricketts CJ, De Cubas AA, Fan H, Smith CC, Lang M, Reznik E, Bowlby R, Gibb EA, Akbani R, Beroukhim R, et al: **The Cancer Genome Atlas Comprehensive Molecular Characterization of Renal Cell Carcinoma.** *Cell Rep* 2018, **23:**313-326 e315.

2. Gordan JD, Lal P, Dondeti VR, Letrero R, Parekh KN, Oquendo CE, Greenberg RA, Flaherty KT, Rathmell WK, Keith B, et al: **HIF-alpha effects on c-Myc distinguish two subtypes of sporadic VHL-deficient clear cell renal carcinoma.** *Cancer Cell* 2008, **14:**435-446.

3. International Cancer Genome C, Hudson TJ, Anderson W, Artez A, Barker AD, Bell C, Bernabe RR, Bhan MK, Calvo F, Eerola I, et al: **International network of cancer genome projects.** *Nature* 2010, **464:**993-998.

4. Motzer RJ, Banchereau R, Hamidi H, Powles T, McDermott D, Atkins MB, Escudier B, Liu LF, Leng N, Abbas AR, et al: **Molecular Subsets in Renal Cancer Determine Outcome to Checkpoint and Angiogenesis Blockade.** *Cancer Cell* 2020, **38:**803-817 e804.

5. Tate JG, Bamford S, Jubb HC, Sondka Z, Beare DM, Bindal N, Boutselakis H, Cole CG, Creatore C, Dawson E, et al: **COSMIC: the Catalogue Of Somatic Mutations In Cancer.** *Nucleic Acids Res* 2019, **47:**D941-D947.

6. Cheng C, Yan X, Sun F, Li LM: **Inferring activity changes of transcription factors by binding association with sorted expression profiles.** *BMC Bioinformatics* 2007, **8:**452.

7. Thorsson V, Gibbs DL, Brown SD, Wolf D, Bortone DS, Ou Yang TH, Porta-Pardo E, Gao GF, Plaisier CL, Eddy JA, et al: **The Immune Landscape of Cancer.** *Immunity* 2018, **48:**812-830 e814.

8. Li T, Fan J, Wang B, Traugh N, Chen Q, Liu JS, Li B, Liu XS: **TIMER: A Web Server for Comprehensive Analysis of Tumor-Infiltrating Immune Cells.** *Cancer Res* 2017, **77:**e108-e110.

9. Nirmal AJ, Regan T, Shih BB, Hume DA, Sims AH, Freeman TC: **Immune Cell Gene Signatures for Profiling the Microenvironment of Solid Tumors.** *Cancer Immunol Res* 2018, **6:**1388-1400.

10. Gu Z: **Complex heatmap visualization.** *Imeta* 2022, **1:**e43.

11. Subramanian A, Tamayo P, Mootha VK, Mukherjee S, Ebert BL, Gillette MA, Paulovich A, Pomeroy SL, Golub TR, Lander ES, Mesirov JP: **Gene set enrichment analysis: a knowledge-based approach for interpreting genome-wide expression profiles.** *Proc Natl Acad Sci U S A* 2005, **102:**15545-15550.

12. Hanzelmann S, Castelo R, Guinney J: **GSVA: gene set variation analysis for microarray and RNA-seq data.** *BMC Bioinformatics* 2013, **14:**7.
